# Southern Carpathian ultramafic grasslands within the central-southeast European context: syntaxonomic classification and overall eco-coenotic patterns

**DOI:** 10.1186/s40529-022-00355-8

**Published:** 2022-10-12

**Authors:** Gheorghe Coldea, Dan Gafta, Gavril Negrean, Adrian Ilie Stoica, Bogdan-Iuliu Hurdu

**Affiliations:** 1 Institute of Biological Research, National Institute for Research and Development in Biological Sciences, 48 Republic Street, Cluj-Napoca, Romania; 2 Department of Taxonomy and Ecology and Centre 3B, Babeș-Bolyai University, 42 Republic Street, Cluj-Napoca, Romania; 3 Dimitrie Brândză Botanic Garden, 32 Cotroceni Road, Bucharest, Romania

**Keywords:** Balkan Peninsula, Elevation, Heavy metals, New syntaxa, Serpentine habitats, Species richness, Soil pH, Southern Carpathians, Terrain slope, Vegetation cover

## Abstract

**Background:**

Previous investigations carried out in ultramafic habitats emphasized the greater importance of site conditions over soil toxic metal content for vegetation composition. Very little is known about the floristic structure of the Southern Carpathian ultramafic grasslands and there is no information on the local environmental drivers of their composition and coenotic features. Here, we aim to fill these knowledge gaps by referring to similar phytocoenoses described in the Balkan Peninsula and central Europe. In particular, we searched for: (i) floristic and ecological patterns supporting the classification and taxonomic assignment of these grasslands, and (ii) simple relationships between serpentine vegetation characteristics and its physiographic environment. A total of 120 phytosociological relevés, of which 52 performed in the Southern Carpathians, were analysed through cluster, ordination and regression procedures.

**Results:**

Despite some floristic similarities with their Balkan counterparts, the Southern Carpathian ultramafic grasslands were clustered into four distinct groups, which were assigned to as many new syntaxa: *Plantago serpentinae–Armerietum halleri*, *Asplenio serpentini–Achnatheretum calamagrostis*, *Minuartio frutescentis–Plantaginetum holostei* and *Sileno saxifragae–Plantaginetum holostei*. The latter was best individualised through the occurrence of several Carpathian endemic taxa. The first two ordination axes were significantly related with the terrain slope/presence of xerophilous species and respectively, with site elevation/presence of calcifugous species. The total plant cover showed a unimodal relationship with respect to site elevation. While controlling for the effect of the sampled area, species richness showed a unimodal response to both elevation and slope of the terrain, although their effects were not singular.

**Conclusions:**

The syntaxonomic distinctiveness of the Southern Carpathian ultramafic grasslands is mainly supported by their overall species composition rather than regional differential species. The main limiting factors driving the composition, cover and species richness of all studied ultramafic grasslands are the water deficit at low elevation and on steep slopes, and the low soil fertility at higher elevations. Our results confirm the previous findings according to which physiographic conditions and, to a lesser extent, soil base nutrients are more important than heavy metal concentrations in structuring the ultramafic vegetation.

**Supplementary Information:**

The online version contains supplementary material available at 10.1186/s40529-022-00355-8.

## Background

The particularities of the ultramafic flora and vegetation have raised much interest from scientists all over the world (Baker et al. 1992; Roberts and Proctor 1992). Petrographic materials that exhibit a low Ca:Mg ratio (< 1), high amount of Mg and Fe, as well as high concentration of Ni, Cr and Co are referred to as ultramafic rocks (Kruckeberg 2002; Alexander et al. 2007). Depending on their origin, igneous or metamorphic, two major types of such rocks can be distinguished: peridotite and respectively, serpentinite (Kierczak et al. 2021). The former tend to have higher Fe-oxide concentrations than the latter (Alexander and DuShey 2011).

Ultramafic soils developed on such substrates generally have poor fertility, given the low macronutrient (N, P, K, Ca) content, which is due to both bedrock properties and low input of dead organic matter from the sparse vegetation (Kruckeberg 1985; Brooks 1987; Robinson et al. 1997; Chiarucci et al. 1998a, b; Proctor 2003). In addition, these soils are susceptible to drought because of their coarse texture, rockiness and shallowness (Brooks 1987; Robinson et al. 1997; Kruckeberg 2002). Nevertheless, a large variation in terrain slope and local climate can lead to a divergence of edaphic characteristics from those of the underlying ultramafic rocks (D’Amico et al. 2014). Such a case may occur under conditions of a humid climate on plateaus/mild slopes where rock weathering and cation leaching is enhanced, eventually translating in slightly deeper, less skeletal but more acidic soils (Chardot et al. 2007; D’Amico and Previtali 2012).

Ultramafic habitats usually host specialist plant taxa (serpentinophytes) that display specific adaptations to the harsh edaphic conditions (Kruckeberg 1985; Brooks 1987; Batianoff and Singh 2001; Chiarucci 2003; Stevanović et al. 2003). The so-called 'serpentine syndrome' (Jenny 1980) is mainly reflected in reduced biomass productivity and stress/toxicity displayed by the generalist species. Not surprisingly, the plant assemblages established on ultramafic soils are compositionally distinct from adjacent communities developed on non-ultramafic soils (Brooks 1987; Baker et al. 1992; Robinson et al. 1996; D’Amico et al. 2014; El Ghalabzouri et al. 2015) and generally display lower relative cover due to the negative effects of high Ni content (Lee 1992; Chardot et al. 2007). However, Ni availability appears to be a minor driver of vegetation composition in serpentine areas, while playing a major role in discriminating between ultramafic and non-ultramafic plant communities (D’Amico et al. 2014). Several studies showed that topo-climatic conditions, such as slope, elevation and solar heat load, or fertility-related factors, like soil rockiness and base nutrient content, have a greater influence on plant species composition than high concentration levels of heavy metals (Chiarucci et al. 1998a, 1998b, 2001; Tsiripidis et al. 2010; D’Amico et al. 2014; El Ghalabzouri et al. 2015). Furthermore, plant species richness in ultramafic habitats seems to be mainly controlled by other factors (e.g., soil moisture) than the toxic metal content (Reddy et al. 2009; Brković et al. 2015).

Ultramafic bedrocks within the Southern Carpathian range have a patchy distribution, being restricted to small patches underlying shallow, skeletal soils in only four mountain groups: the Almăjului, Mehedinți, Retezat and Cozia Mountains (Rădulescu and Dumitrescu 1966). The few vegetation studies dealing with such particular habitats were limited to and only linked with the presence of a rare serpentinophytic species in Romania i.e., *Plantago holosteum* (Boșcaiu et al. 1974; Coldea and Pop 1988). In previous floristic studies covering the Mehedinți and Almăjului Mts. (Ciortan and Negrean 2012; Negrean and Ciortan 2012a, b), the occurrence of a series of plant taxa (i.e., *Armeria alpina* subsp. *halleri*, *Asplenium serpentini*, *Dorycnium pentaphyllum* subsp. *germanicum*, *Euphrasia illyrica*, *Notholaena marantae*, *Plantago serpentina*, *Potentilla cinerea* subsp. *tommasiniana* and *Silene bupleuroides*), known for their preference for serpentine-rich substrates, was documented. As the above-mentioned taxa are widely distributed in the Balkan Peninsula, certain floristic similarities between the ultramafic herbaceous communities from the Southern Carpathians and their counterparts from the western and the southern Balkan Peninsula are noticeable, the latter being assigned to the orders *Halacsyetalia* (Ritter-Studnička 1970; Aćić et al. 2015; Kuzmanović et al. 2016) and respectively, *Trifolietalia parnassi* (Raus 1987). On the other hand, the southern Balkan ultramafic communities from Greece differ physiognomically by far from those developed on similar bedrocks in Bulgaria and assigned to the order *Astragalo–Potentilletalia* (Tzonev et al. 2013). Consequently, a more in-depth investigation of the floristic structure of the Southern Carpathian ultramafic grasslands and their differentiation from their Balkan counterparts is required, in order to assess the adequate syntaxonomic assignation of the former. For this purpose, we also considered ultramafic, central-European herbaceous communities that were classified in the order *Violetalia calaminariae* (Ernst 1965, 1974).

Therefore, we hereby aim to assess: (i) the proper syntaxonomic assignation of the herbaceous communities developed on ultramafic rocks in the Southern Carpathians and their phytogeographic particularities in a wider geographical context; (ii) the ecological ordination of all considered, central and south-eastern European coenoses along edaphic and topoclimatic gradients; and (iii) the strength and shape of the relationships between total species cover/richness and local topographic variables by jointly considering all studied communities.

## Materials and methods

### Study areas

**Fig. 1:**
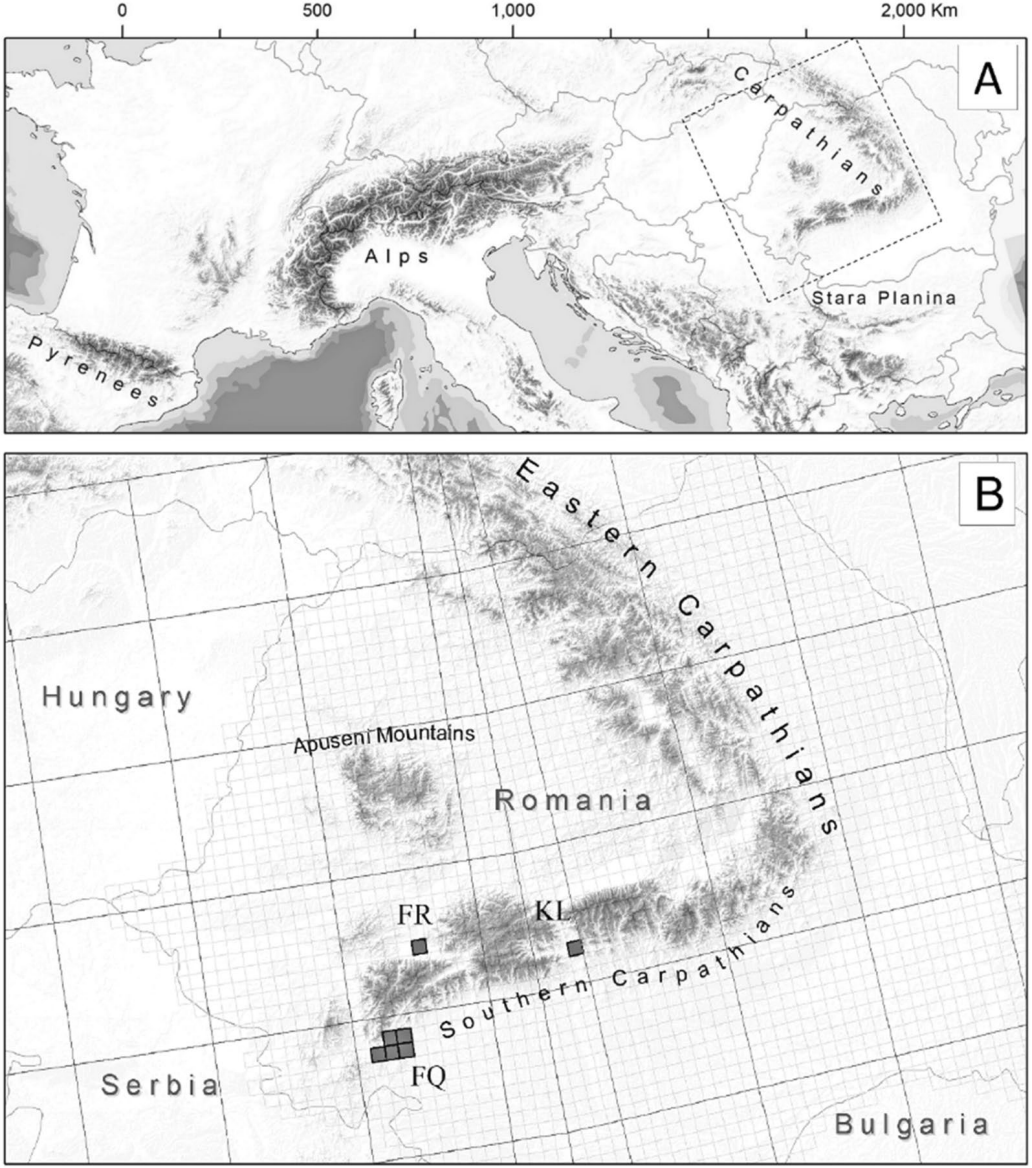
Location of the Carpathians within Europe (**A**) and of the study sites within the Southern Carpathians (**B**) by reference to the 10 × 10 km UTM-grid cells (FQ = Mehedinți Mts.; FR = Retezat Mts.; KL = Cozia Mts.)

The Southern Carpathians, one of the four main subunits of the Carpathian Mountains (that include the Western and Eastern Carpathians, and the Apuseni Mountains), lie within the Central European floristic region (Fig. 1A). Their south-western edge, encompassing the Mehedinți Mountains (Fig. 1B), is limitrophe to the Submediterranean region (Frey and Lösch 2010) and therefore, shares some climatic and biotic characteristics with the neighbouring mountains of the Balkan Peninsula. The mean annual temperatures in the Mehedinți Mts. range between 7.2 and 10.1 °C (Szalai et al. 2013), while the annual precipitations, which amount on average to about 800 mm (655–900 mm), are unevenly distributed throughout the year, determining a pronounced water deficit in August (Roman 1974). The other two disjunct study areas, located in the Retezat and Cozia Mountains (Fig. 1B), are overall characterised by a slightly cooler climate, but the investigated sites benefit from milder temperatures due to their sunny exposure and, in some cases, low elevations. Annual mean temperatures range between 3 °C (in the Cozia Mts.) and 7 °C (the Poieni Peak, in the Retezat Mts.), while annual precipitations sum up to 800–950 mm.

The areas featuring ultramafic bedrocks in the Southern Carpathians are restricted to small, scattered patches of 3000–15,000 m^2^, embedded within a platform of crystalline schists. The overlying ultramafic soils are poorly developed (15–20 cm deep), usually rich in detritus and attributable to either rendzinic or pararendzinic leptosols, depending on the absence or respectively, the presence of remnant disturbance due to mining activities (Florea and Munteanu 2003).

### Data collection

The delimitation of areas featuring ultramafic substrates was based on existing geological maps at the scale 1:200,000 (Bleahu et al. 1968). The selected study sites were initially mapped using the UTM grid system with 10 × 10 km sized quadrats (Fig. 1B). Larger areas covered with specific serpentine vegetation were found in the Mehedinți Mts. at altitudes varying between 350 and 1100 m (FQ quadrats in Fig. 1B). Additional, smaller areas were spotted in the Retezat Mts. at 470 m (quadrat FR in Fig. 1B) and in the Cozia Mts. at 1500 m (quadrat KL in Fig. 1B).

A reference list of plant taxa, which occur preferentially on ultramafic substrates (serpentinophytes), was compiled on the basis of several published studies performed in the Balkan Peninsula or in central Europe (e.g., Stevanović et al. 2003; Dierschke and Becker 2008; Jakovljević et al. 2011; Kuzmanović et al. 2016).

The vegetation survey was carried out in 2017–2018 using the phytosociological method (Braun-Blanquet 1964). The relevé plots were placed on the basis of the presence of serpentinophytes. The relevé area varied between 4 and 10 m^2^, being constrained by the local site conditions. Except for bryophytes, which were largely missing due to the relative high proportion of gravel (40*–*50% on average), all the occurring plant species were recorded by visually estimating their abundance on the Braun-Blanquet ordinal scale. The topographic characteristics of the sampled habitats, such as aspect and slope, were collected using an Eclipse 99 compass & clinometer, while the coordinates were registered using a GPS unit (Garmin GPSMAP 64S). All the basic information regarding the geo-topographical characteristics of the investigated sites, along with the number of relevés performed in each of them, are reported in Table 1.

**Table 1:** Geo-topographical characteristics of the sites investigated in the Southern Carpathians

Site name (toponym)	No. of relevés	UTM cell code	Latitude (degrees)	Longitude (degrees)	Elevation (m)	Aspect
Ciolanu Mare	17	FQ-27	44.93330	22.51821	1050	W-SW
Ciolanu Mic	2	FQ-28	44.93914	22.52472	1030	SW
Dealul Comorişte	3	FQ-27	44.87616	22.49125	890	S
Dealul cu Zgură	1	FQ-38	45.01949	22.69503	471	W
Valea Verde	1	FQ-38	45.00912	22.65822	610	NW
Valea Coşuştei	2	FQ-28	44.97028	22.57545	570	S
Rudina	6	FQ-47	44.88551	22.78273	400	S-SW
Cozia Mts.	10	KL-82	45.31768	24.33872	1540	SW
Vf. Poieni	10	FR-54	45.55178	22.97258	420	SE

Subsequent soil sampling was carried out in 2020 after revisiting two to four representative sites, in terms of floristic composition, for each plant community type. One soil sample per site was collected from the mineral topsoil (1–12 cm deep) and subsequently analysed using an atomic absorption spectrophotometer, model novAA350 (Analytic Jena). The pH and heavy metal content of the soil samples are presented in Table 2. The dominant ultramafic rock type (serpentinites or peridotites) was determined on the basis of soil samples (Table 2) or, when these were not available, by employing geological maps.

**Table 2:** Heavy metal content (mg/kg) and pH of soil samples collected in the investigated ultramafic sites from the Southern Carpathians

Site name	Plant association	Elevation (m)	Ultramafic rock type	pH	Fe	Cd	Cu	Ni	Zn	Cr	Pb	Co	Mn	Mg
Mehedinți Mts.														
Ciolanu Mare	PA	1130	ser	7.3	137.0	3.0	32.6	1564.7	59.9	330.9	26.8	103.5	821.7	56.0
Ciolanu Mic	PA	1025	ser	6.5	275.0	3.3	21.7	1511.3	51.3	426.7	9.7	110.6	855.6	72.9
Obârșia Cloșani	AsA	471	ser	6.1	193.0	2.9	21.9	1538.2	43.9	335.2	11.4	100.3	913.8	43.9
Giurgieni—Valea Coșutei	AsA	567	ser	6.5	325.0	3.5	19.9	1538.9	41.2	507.1	8.1	103.8	1115.6	30.4
Rudina	AsA	405	ser	6.4	275.0	3.3	21.7	1511.3	51.3	426.7	9.7	110.6	855.6	58.7
Cozia Mts.														
Pereții Gradului	SP	1520	ser	5.3	141.0	2.2	26.8	1373.0	44.1	331.0	29.7	98.7	765.2	47.3
Ciuha Mică	SP	1546	ser	4.8	198.0	2.7	22.7	1389.6	26.8	354.8	8.8	97.5	744.1	45.2
Ciuha Mare	SP	1589	ser	4.1	210.0	3.1	19.7	1435.7	39.1	345.2	10.7	101.8	874.1	35.1
Retezat Mts.														
Vf. Poienii	MP	432	per	5.1	625.0	4.9	15.3	10.6	51.1	20.7	29.7	10.2	228.7	60.8
Vf. Poienii	MP	410	per	4.4	537.0	4.2	15.4	13.7	58.4	19.5	33.9	9.8	261.7	43.6
Vf. Poienii	MP	400	per	4.3	525.0	4.5	16.5	14.5	50.9	21.9	20.2	9.7	226.6	35.8
Vf. Poienii	MP	385	per	4.8	181.0	5.3	15.4	9.5	67.1	21.1	41.4	11.4	306.7	18.2

ser, serpentinites; per, peridotites; PA, *Plantago serpentinae–Armerietum halleri*; AsA, *Asplenio serpentini–Achnatheretum calamagrostis*; SP, *Sileno saxifragae–Plantaginetum holostei*; MP *Minuartio frutescentis–Plantaginetum holostei*

The phytosociological data, not pertaining to Southern Carpathians, were retrieved from the literature as follows: 26 relevés of *Armerietum halleri* from Harz Mts. in central Germany (Dierschke and Becker 2008), 12 relevés of *Poo molinerii–Plantaginetum holostei* from Mt. Studena Planina in central Serbia (Tatić 1969), 5 relevés of *Artemisio albae–Achnatheretum calamagrostis* from Mt. Kopaonik in southern Serbia (Jovanović et al. 2017), 16 relevés of *Onosmo–Festucetum dalmaticae* from eastern Rhodope Mts. in Bulgaria (Tzonev et al. 2013), and 9 relevés of *Anthemido–Plantaginetum holostei* from Mt. Ossa in central Greece (Raus 1987). A synthetic table including the species frequencies of occurrence in all considered plant associations is presented in Additional file 1: Appendix S1.

The taxonomic nomenclature used for recording the plant species followed the Euro + Med (2006) PlantBase, with few exceptions regarding some specialised taxa with limited distribution (e.g., *Anthemis cretica* subsp. *kitaibelii*, *Potentilla cinerea* subsp. *tommasiniana, Pilosella hoppeana* subsp. *testimonialis*), for which we prioritised the taxonomic view expressed by the regional flora (Horvat et al. 1974; Sârbu et al. 2013).

### Data analysis

Prior to numerical analyses, the species abundance scores were converted into presence-absence values. We chose this approach to better distinguish community types based on phytogeographical diagnostic taxa, regardless of their abundance. The classification of the 120 relevés based on their pairwise Sørensen dissimilarities was performed via hierarchical cluster analysis by using different algorithms (average linkage, beta-flexible and Ward). The dendrogram output by the former method was eventually retained as it delivered the highest cophenetic correlation coefficient (0.929), i.e. the smallest distortion of the input floristic dissimilarities. The optimal number of clusters was determined based on agreement among four criteria (average silhouette width, Dunn coefficient, Calinski-Harabasz index, and prediction strength) in terms of the location of the maximum value among those corresponding to all possible solutions with 2–12 clusters. The stability of each retained cluster (expressed in percentages) was assessed by bootstrapping the mean Jaccard similarity of the component relevés. In order to assist in the distinction of diagnostic species of single or groups of syntaxa, the group-equalized Phi coefficient was used to test the association strength and significance between the retained groups of relevés and each individual species.

Local, non-metric multidimensional scaling (NMDS) applied on the same (dissimilarity) input matrix was employed for indirect ordination of relevés in the species space. Ecological gradients related to the NMDS axes were partly inferred with the aid of Ellenberg’s indicator values of species displaying the largest ordination scores (Ellenberg et al. 2001; Sârbu et al. 2013). In addition, the available topographic variables (elevation and slope) were tested separately as dependent variables against the three extracted NMDS axes by linear trend surface fitting.

Polynomial, simple or multiple regressions were employed to test the non-linear dependency of total plant cover or species richness on topographic variables by jointly considering all studied communities. Both response variables and predictors were either square-rooted or log-transformed to reduce skewness and heteroscedasticity. In addition, all independent variables were centered to reduce multicollinearity in multiple regressions.

All analyses were carried out in the R software environment, using several packages: *stats* (R Core Team 2021), *vegan* (Oksanen et al. 2020), *cluster* (Maechler et al. (2021), *fpc* (Hennig 2020) and *indicspecies* (De Cáceres et al. 2020). The maps were generated using ArcGIS 9.3.1 (ESRI 1999–2009).

## Results

### Classification of all relevés

**Fig. 2:**
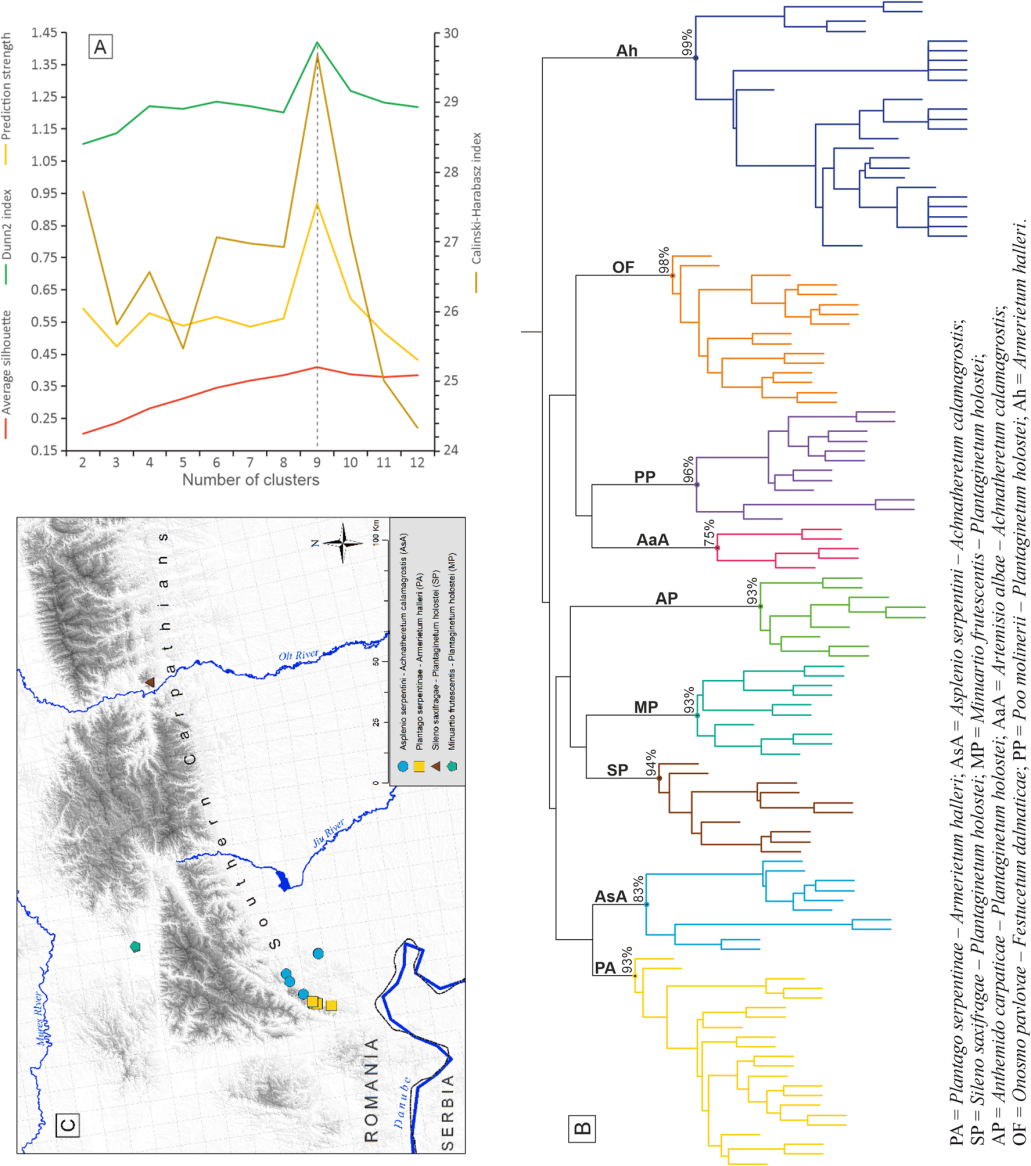
**A** Optimal number of clusters determined by consensus among the maxima of four validation criteria calculated for all possible solutions ranging from 2 to 12 clusters; **B** Output dendrogram of the cluster analysis performed on the matrix of compositional dissimilarities between the 120 relevés under study (the percentage values indicate the stability of the nine retained clusters); **C** Distribution of the four Southern Carpathian herbaceous associations distinguished on ultramafic substrates

The distributions of the four validation criteria as a function of cluster counts point jointly to an optimal classification of the 120 relevés in nine clusters (Fig. 2A). All of them have a relatively high stability that varied between 75 and 99% (Fig. 2B). The smallest cluster, encompassing the five communities of *Artemisio albae–Achnatheretum calamagrostis* (AaA), shows the lowest stability (Fig. 2B). All the relevés from the Balkan Peninsula and central Europe originally assigned to *Anthemido–Plantaginetum holostei* (AP), *Artemisio albae–Achnatheretum calamagrostis* (AaA), *Poo molinerii–Plantaginetum holostei* (PP), *Onosmo–Festucetum dalmaticae* (OF) and *Armerietum halleri* (Ah) were included in separate clusters matching the previously mentioned plant associations (Fig. 2B). The relevés pertaining to the Southern Carpathians were grouped into the remaining four clusters (Fig. 2B).

### Syntaxonomic assignation of the Southern Carpathian relevés

The four clusters of Southern Carpathian relevés were syntaxonomically associated with as many new plant associations (Fig. 2B and Tables 4, 5, 6, 7): *Plantago serpentinae*–*Armerietum halleri* (PA), *Asplenio serpentini–Achnatheretum calamagrostis* (AsA), *Sileno saxifragae–Plantaginetum holostei* (SP) and *Minuartio frutescentis*–*Plantaginetum holostei* (MP). Within the context of the 120 relevés analysed, each of the four Southern Carpathian ultramafic grassland communities stands out through at least two statistically significant, discriminant species (Table 3).

**Table 3:** Discriminant species for each syntaxon distinguished in the Southern Carpathians within the context of all studied ultramafic grasslands (only species with significant Phi coefficients larger than 0.75 are listed)

Syntaxa discriminated/Species names	Phi	p-value
***Plantago serpentinae–Armerietum halleri***		
*Plantago serpentina*	0.921	0.0001
*Potentilla cinerea* subsp. *tommasiniana*	0.893	0.0001
***Asplenio serpentini–Achnatheretum calamagrostis***		
*Alyssum petraeum*	0.821	0.0001
*Asplenium serpentini*	0.803	0.0001
***Sileno saxifragae–Plantaginetum holostei***		
* Silene saxifraga*	0.883	0.0001
* Festuca saxatilis*	0.883	0.0001
* Pilosella rhodopea*	0.756	0.0001
* Luzula luzuloides*	0.756	0.0001
* Silene lerchenfeldiana*	0.756	0.0001
***Minuartio frutescentis–Plantaginetum holostei***		
* Anthemis cretica* subsp. *kitaibelii*	0.821	0.0001
* Minuartia hirsuta* subsp. *frutescens*	0.817	0.0001

In accordance with their grouping within the dendrogram (Fig. 2B) and the frequencies of occurrence of the characteristic species for the upper syntaxa (Additional file 1: Appendix S1), PA and AsA, on one side and, SP and MP, on the other side, were assigned to different alliances and orders within the class *Festuco–Brometea*. The complete syntaxonomical scheme, encompassing the four ultramafic grassland types (plant associations) distinguished in the Southern Carpathians, is shown below.

Class *Festuco–Brometea* Br.-Bl. et Tx. ex Soó 1947

 Order *Halacsyetalia sendtneri* Ritter-Studnička 1970

      Alliance *Thymion jankae* (Kojić et al.) *ex* Coldea et al. all. nova hoc loco

*         Plantago serpentinae–Armerietum halleri* ass. nova hoc loco

*          Asplenio serpentini–Achnatheretum calamagrostis* ass. nova hoc loco

 Order *Stipo pulcherrimae–Festucetalia pallentis* Pop 1968

      Alliance *Asplenio septentrionalis–Festucion pallentis* Zolyomi 1936 corr. 1966

*         Sileno saxifragae–Plantaginetum holostei* ass. nova hoc loco

*         Minuartio frutescentis*–*Plantaginetum holostei* ass. nova hoc loco

### Diagnosis of the validated alliance Thymion jankae

Name-giving species: *Thymus praecox* subsp. *jankae* (Čelak.) Jalas

Nomenclature type (holotypus): *Poo alpinae–Plantaginetum holostei* Kojić et Ivanović 1953

Diagnostic taxa: *Asplenium* *serpentini*, *Thymus praecox* subsp. *jankae*, *Armeria alpina, Alyssum murale* subsp. *pichleri, Plantago serpentina* and, exclusively in the Dinarides, *Viola macedonica, Euphorbia serpentini, Bornmuellera dieckii.*

Habitat: xerophilous, open grasslands developed on shallow, neutral soils overlying ultramafic substrates (either as consolidated rocks or fine-grained screes), from colline to lower montane belt (300–1100 m a.s.l.)

Distribution: eastern range of the central Dinarides (e.g., Maljen, Zlatibor, Studena and Ozren Mts. in Serbia) and south-western end of the Southern Carpathians (i.e., Mehedinți and Almăjului Mts. in Romania).

### Floristic and habitat characteristics of the Southern Carpathian ultramafic communities

***Plantago serpentinae–Armerietum halleri*** ass. nova hoc loco

Holotypus: relevé 13 (Table 4); Abbreviation: PA; Photo: B and C in Additional file 2: Appendix S2.

**Table 4:** *Plantago serpentinae–Armerietum halleri* ass. nova (* holotypus; ser—serpentinites)

Relevé no.	1	2	3	4	5	6	7	8	9	10	11	12	13*	14	15	16	17	18	19	20	24	25
Aspect	SW	W	W	N	S	E	E	S	S	-	S	SW	SW	E	SW	W	W	S	S	E	-	-
Slope (degrees)	5	15	30	5	5	5	5	5	5	+	10	10	5	10	5	15	15	5	5	5	+	+
Elevation (m)	1020	1030	1050	1050	1000	1100	1130	1070	1100	1080	1100	1080	1080	1080	1060	1010	1010	890	870	870	1030	1040
Total herb cover (%)	50	75	55	90	60	80	75	60	60	70	55	60	60	50	60	75	65	70	65	85	85	65
Plot area (m^2^)	10	10	10	10	10	10	10	10	10	10	10	10	10	10	10	10	10	10	10	10	10	10
Bedrock type	ser	ser	ser	ser	ser	ser	ser	ser	ser	ser	ser	ser	ser	ser	ser	ser	ser	ser	ser	ser	ser	ser
**Diagnostic species at association level**																						
Armeria alpina subsp. halleri	1.3	2.3	2.3	1.2	+.1	+.2	2.3	1.2	2.3	1.3	1.2	1.2	1.2	+.2	+.1	1.2	+.1	+.2	+.1	3.4	2.3	3.4
Plantago serpentina	2.3	2.3	3.4	2.3	2.2	.	+	.	.	3.3	2.3	2.2	3.4	2.3	2.2	+.1	2.3	+	+	+	3.4	2.3
**Thymion jankae and Halacsyetalia**																						
Potentilla cinerea subsp. tommasiniana	+	1.2	.	2.3	2.2	.	3.4	2.3	3.4	1.2	3.3	1.2	2.3	2.2	+	3.4	+	2.3	2.4	+	+	+
Dorycnium pentaphyllum subsp. germanicum	1.2	3.4	1.2	+	.	.	2.3	3.3	.	.	.	+	+	+	.	2.2	3.4	+	+	+	.	+
Silene bupleuroides subsp. staticifolia	+	1.2	1.3	1.2	.	.	+	+	+	.	.	+	.	+	+	2.3	+	+	+	+	.	2.3
Scleranthus perennis subsp. dichotomus	+	.	.	.	1.2	1.2	.	.	+	.	.	.	.	.	.	.	.	.	1.2	1.2	+.2	.
Thymus praecox subsp. jankae	+	.	.	+	.	.	1	.	+	.	+	.	+	.	1	.	+	1	+	1	.	.
Dianthus giganteus subsp. banaticus	.	+	.	+	.	+	+	.	.	.	.	.	+	.	.	+	.	+	+	+	.	.
Asplenium serpentini	.	.	.	.	.	.	.	.	.	.	.	3.3	2.3	2.2	3.4	2.3	2.4	.	.	.	.	.
Pilosella hoppeana subsp. testimonialis	.	.	.	.	3.4	+	.	.	.	1.2	.	.	.	.	.	.	.	.	.	.	.	.
Alyssum murale subsp. pichleri	.	.	.	.	.	.	.	.	.	.	.	.	.	.	.	.	.	2.3	2.3	+	.	.
Bromus riparius	.	.	.	.	+	.	.	+	.	.	.	.	.	.	.	.	.	.	.	.	.	.
Notholaena marantae	.	.	.	.	.	.	.	.	.	.	.	.	.	.	.	.	.	3.5	3.5	.	.	.
**Festuco–Brometea**																						
Thymus praecox subsp. polytrichus	1.2	+	.	+	+	.	2.2	.	+	.	+	+	+	.	2.3	+	+	2.3	+	2.2	.	.
Pilosella pavichii	.	1.2	+.2	.	.	.	+	+	1.2	.	+	+	+	.	+	+	+	.	+	.	1.2	.
Festuca valesiaca	2.3	2.3	.	.	+	+	2.2	2.2	1.2	.	+	1.3	+	.	+	.	.	2.2	1.2	.	.	.
Danthonia alpina	+	.	+	1.1	.	.	.	.	.	.	+	.	.	+	2.3	1.2	+	+	.	.	.	.
Koeleria macrantha	+	.	+	1.2	1.3	+	.	+	+.1	.	+	.	.	.	.	.	+	.	+	.	+	+
Achillea pannonica	.	+	.	+	.	+	+	.	.	.	.	.	.	+	.	.	+	.	.	+	+	+
Veronica orchidea	.	+.2	.	+	.	.	+	.	.	.	.	.	+	+	+	.	.	.	.	.	.	+
Minuartia hirsuta subsp. frutescens	.	+	+	.	.	.	.	1.1	.	.	.	.	.	.	.	.	2.3	+	+	+	.	.
Galium verum	.	.	.	+	.	+	+	.	.	.	+	.	.	.	.	+	.	.	.	.	+	.
Achillea crithmifolia	.	.	.	.	.	.	.	.	.	+	.	.	.	.	+	+	.	+	.	+	.	.
Festuca pallens	+	.	.	.	.	.	.	.	.	.	.	.	+	+	.	.	.	.	.	.	+	.
Melica ciliata	.	.	.	.	.	3.3	.	.	+	.	.	.	.	.	.	.	.	.	+	.	.	.
Asperula cynanchica	.	+	.	.	.	.	.	.	.	.	.	.	.	.	.	.	+	.	+	.	.	.
Hypericum perforatum	.	.	.	.	.	.	.	.	+	.	+	.	.	+	.	.	.	.	.	.	.	.
Sedum hispanicum	.	.	.	.	.	+	.	+	.	.	.	.	.	.	.	.	.	.	.	.	.	.
Asyneuma canescens	.	.	.	.	.	.	+	.	.	.	.	.	.	.	.	.	+	.	.	.	.	.
Scabiosa columbaria	.	.	.	.	.	.	.	.	+	.	.	+	.	.	.	.	.	.	.	.	.	.
Petrorhagia saxifraga	.	.	.	.	.	.	.	.	.	.	.	.	.	.	.	.	.	+	.	+	.	.
Eryngium campestre	.	.	.	.	.	.	.	.	.	.	.	.	.	.	.	.	.	.	+	+	.	.
Carex humilis	.	.	.	+	.	.	.	.	.	.	.	.	.	.	.	.	.	.	.	.	.	.
Prunella laciniata	.	.	.	.	.	.	.	.	.	.	.	.	+	.	.	.	.	.	.	.	.	.
Silene armeria	.	.	.	.	.	.	.	.	.	.	.	.	.	+	.	.	.	.	.	.	.	.
Teucrium chamaedrys	.	.	.	.	.	.	.	.	.	.	.	.	.	.	.	+	.	.	+	+	.	.
Bromus erectus	.	.	.	.	.	.	.	.	.	.	.	.	.	.	.	.	.	+.2	.	.	.	.
Allium flavum	.	.	.	.	.	.	.	.	.	.	.	.	.	.	.	.	.	.	+	.	.	.
Euphorbia cyparissias	.	.	.	.	.	.	.	.	.	.	.	.	.	.	.	.	.	.	+	.	.	.
Centaurea stoebe subsp. australis	.	.	.	.	.	.	.	.	.	.	.	.	.	.	.	.	.	.	.	+	.	.
**Companions**																						
Anthoxanthum odoratum	.	+	.	+	+	1.2	1.2	+	1.2	2.3	+.2	+	1.2	+	.	+	+	.	.	+	2.3	+
Agrostis capillaris	+	+	+	1.3	+	2.2	+	.	+	.	.	.	.	+	+	+	.	.	.	.	2.3	.
Galium album	.	.	+	.	.	+	+	+	1.2	.	.	.	.	+	.	+	+	.	.	.	.	.
Cerastium pumilum subsp. glutinosum	.	+	.	.	.	.	.	.	.	.	.	+	.	+	+	.	.	+	+	+	.	.
Poa alpina agg.	.	.	.	.	.	.	.	+	.	+	+.3	+	.	.	.	.	+	.	.	.	+	+
Euphrasia illyrica	.	.	.	.	.	.	.	.	.	.	.	.	+	+	.	+	+	+	+	.	.	.
Festuca ovina	.	.	+	3.4	1.2	.	.	.	.	.	.	.	.	.	.	.	.	.	.	1.3	+	.
Rorippa pyrenaica	.	+	.	.	.	.	.	+	.	.	+	.	.	.	.	.	.	.	.	.	+	.
Luzula campestris	.	.	.	+	+	+	.	.	.	+	.	.	.	.	.	.	.	.	.	.	.	.
Bromus squarrosus	.	.	.	.	.	.	.	+	.	.	.	.	.	.	.	.	.	2.3	+	3.4	.	.
Lotus corniculatus	.	.	.	.	.	.	.	.	+	.	+	.	.	.	.	+	.	.	.	.	.	+
Festuca pratensis	+	.	.	.	.	2.3	.	.	.	.	.	.	.	.	+	.	.	.	.	.	.	.
Deschampsia flexuosa	.	.	+	+	.	.	.	.	.	1.1	.	.	.	.	.	.	.	.	.	.	.	.
Seseli peucedanoides	.	.	+	+	.	.	.	.	.	.	.	.	.	.	.	+	.	.	.	.	.	.
Carlina biebersteinii	.	.	+	.	.	.	.	+	.	.	.	.	.	.	.	+	.	.	.	.	.	.
Trifolium alpestre	.	.	.	+	.	+	+	.	.	.	.	.	.	.	.	.	.	.	.	.	.	.
Plantago lanceolata	.	.	.	.	.	+	+	.	.	.	.	.	+	.	.	.	.	.	.	.	.	.
Genista sagittalis	.	.	.	.	.	+	+	.	.	.	.	.	.	.	.	+	.	.	.	.	.	.
Carduus candicans	.	.	.	.	.	.	.	.	.	.	.	.	.	.	.	.	.	+	+	+	.	.
Poa compressa	+	.	.	.	.	.	.	.	.	.	.	.	.	.	.	.	.	+	.	.	.	.
Carex ovalis	.	.	.	.	+	+	.	.	.	.	.	.	.	.	.	.	.	.	.	.	.	.
Rumex acetosella	.	.	.	.	.	2.3	.	.	.	.	.	.	.	.	.	.	.	.	.	+	.	.
Asplenium ruta-muraria	.	.	.	.	.	.	.	+	.	.	.	.	.	.	.	.	+	.	.	.	.	.
Chondrilla juncea	.	.	.	.	.	.	.	.	.	+	.	.	.	.	.	.	.	.	+	.	.	.
Asplenium septentrionale	.	.	.	.	.	.	.	.	.	+	.	.	.	.	.	.	.	.	+	.	.	.
Asplenium trichomanes	.	.	.	.	.	.	.	.	.	.	.	.	.	+	.	.	+	.	.	.	.	.
Arenaria serpyllifolia	.	.	.	.	.	.	.	.	.	.	.	.	.	.	.	.	.	+	+	.	.	.
Filago arvensis	.	.	.	.	.	.	.	.	.	.	.	.	.	.	.	.	.	+	.	+	.	.
Danthonia decumbens	.	.	.	.	.	.	.	.	.	.	.	.	.	.	.	.	.	.	.	.	+	+

**Companion species with one occurrence:** Achnatherum calamagrostis 1: + ; Holcus lanatus 1: + ; Lactuca serriola 1: + ; Viola arvensis 3: + ; Briza media 4: + ; Gymnadenia conopsea 4: + ; Hypochaeris maculata 5: + ; Veronica chamaedrys 6: + ; Potentilla inclinata 6: + ; Campanula persicifolia 6: + ; Juniperus communis 10: + ; Silene vulgaris 14: + ; Rubus candicans 14: + ; Rhinanthus rumelicus 17: + ; Phleum montanum 18: + ; Brachypodium pinnatum 18: + ; Verbascum phlomoides 19: + ; Trifolium arvense 19: + ; Alyssum alyssoides 20: + ; Cynosurus cristatus 20: + ; Campanula patula 24: +

Relevé sites: 1–17: Mehedinți Mts.—Ciolanu Mare (4.07.2017); 18–20: Dealul Comoriște—Podeni (4.07.2017); 24–25: Ciolanu Mic Mt. (5.07.2017)

These communities develop on undisturbed serpentinite, mild slopes with shallow, rendzina soils (rendzinic leptosol) at altitudes ranging between 870 and 1130 m a.s.l., that is within the limits of the beech (*Fagus sylvatica*) forest belt. The soil samples are characterised by a neutral reaction (pH = 6.8–7.3) with a high content of nickel and chrome (Table 2).

The syntaxonomic assignation of this new plant association was based on the presence of common xerophilous species, characteristic for the *Festuco*–*Brometea* class, and several regional, Balkan or south-European species (i.e., *Notholaena marantae, Alyssum murale* subsp. *pichleri, Silene bupleuroides, Bromus riparius, Asplenium serpentini*). Some acidophilous species (*Deschampsia flexuosa, Asplenium septentrionale, Festuca ovina* and *Luzula campestris*) occur sporadically along with the numerous basiphilous species (Table 4).

*Plantago serpentina* and *Potentilla cinerea* subsp. *tommasiniana* are good discriminant taxa for PA in the context of the studied plant associations (Table 3). In addition, some Anatolic species, like *Pilosella pavichii* and *Pilosella hoppeana* subsp. *testimonialis*, differentiate the PA association from its synvicariant (*Armerietum halleri*) occurring in central Europe (Additional file 1: Appendix S1).

***Asplenio serpentini–Achnatheretum calamagrostis*** ass. nova hoc loco

Holotypus: relevé 26 (Table 5); Abbreviation: AsA; Photo: A in Additional file 2: Appendix S2.

**Table 5:** *Asplenio serpentini–Achnatheretum calamagrostis* ass. nova (* holotypus; ser—serpentinites)

Relevé no.	21	22	23	26*	27	28	29	30	31	32
Aspect	W	S	SW	SE	SE	S	S	NW	W	NW
Slope (degrees)	25	25	30	40	20	5	5	40	10	30
Elevation (m)	470	570	580	340	370	390	410	400	380	610
Total herb cover (%)	55	65	75	55	65	50	50	60	65	50
Plot area (m^2^)	10	10	10	10	10	10	10	10	10	10
Bedrock type	ser	ser	ser	ser	ser	ser	ser	ser	ser	ser
**Diagnostic species at association level**										
Achnatherum calamagrostis	1.3	3.4	4.5	1.1	1.1	1.1	+.1	1.1	+.1	3.3
Asplenium serpentini	1.2	3.4	1.3	+	+	+	+	1.3	+	2.3
Alyssum petraeum	.	.	.	1.3	+	2.3	2.3	+	+	1.2
**Thymion jankae and Halacsyetalia**										
Alyssum murale subsp. pichleri	+	.	.	1.2	1.2	1.1	+	1.3	.	+
Notholaena marantae	2.3	.	.	2.3	3.4	1.3	1.2	2.3	3.4	.
Stachys recta subsp. subcrenata	.	.	.	+.1	+	.	2.2	+	1.3	.
Dorycnium pentaphyllum subsp. germanicum	2.3	1.3	1.3	.	.	.	.	.	.	+
Thymus praecox subsp. jankae	+	+	.	1.3	.	.	.	.	.	+
Dianthus giganteus subsp. banaticus	+	.	.	.	.	.	.	.	.	.
Bromus riparius	.	+	.	.	.	.	.	.	.	.
Potentilla cinerea subsp. tommasiniana	.	.	.	.	.	.	.	.	.	+
**Festuco–Brometea**										
Melica ciliata	.	+	+	1.1	2.3	1.3	1.3	1.3	1.3	+
Pilosella pavichii	+.1	1.2	1.2	+	.	.	.	+	+	+
Festuca valesiaca	.	.	.	2.3	1.3	1.2	1.3	3.4	+	.
Silene armeria	.	+	1.2	+	+	.	.	.	+	.
Asperula cynanchica	.	.	.	.	+	+	+	+	.	+
Thymus praecox subsp. polytrichus	+	+	1.2	.	.	.	.	.	.	1.2
Hypericum perforatum	+	+	.	.	+	.	.	.	.	+
Phleum montanum	.	+	+	+	.	.	.	.	.	+
Allium flavum	.	.	.	1.2	1.3	.	.	+	+	.
Centaurea stoebe subsp. australis	.	+	+	.	.	+	.	.	.	.
Achillea crithmifolia	.	.	.	+	.	.	+	+	.	.
Bothriochloa ischaemum	.	.	.	.	.	+	+	+	.	.
Petrorhagia saxifraga	+	.	.	.	.	+	.	.	.	.
Galium verum	.	+	+	.	.	.	.	.	.	.
Danthonia alpina	+	.	.	.	.	.	.	.	.	.
Sedum hispanicum	+	.	.	.	.	.	.	.	.	.
Festuca pallens	+	.	.	.	.	.	.	.	.	.
Teucrium chamaedrys	.	+	.	.	.	.	.	.	.	.
Eryngium campestre	.	.	.	+	.	.	.	.	.	.
Minuartia hirsuta subsp. frutescens	.	.	.	.	.	.	.	.	.	+
**Companions**										
Rumex acetosella	+	.	.	+	.	+	.	+	+	+
Cynodon dactylon	.	.	.	+	+	+	+	+	1.2	.
Thymus pulegioides	.	.	.	+	+	2.2	.	+	2.2	.
Scleranthus perennis	.	.	.	.	.	+	+	1.2	+	+
Bromus squarrosus	.	+	+	.	+	.	+	.	.	.
Quercus dalechampii	+	.	.	.	.	.	.	.	+	+
Trifolium arvense	.	.	.	.	+	+	.	.	+	.
Cichorium intybus	.	.	.	.	+	+	.	.	+	.
Fraxinus ornus	+	+	.	.	.	.	.	.	.	.
Galium album	+	.	.	+	.	.	.	.	.	.
Moehringia pendula	+	.	.	.	.	.	.	.	.	+
Cerastium pumilum subsp. glutinosum	.	+	+	.	.	.	.	.	.	.
Centaurium erythraea	.	+	+	.	.	.	.	.	.	.
Medicago falcata	.	.	.	.	+	+	.	.	.	.
Filago arvensis	.	.	.	.	.	+	+	.	.	.
Verbascum phlomoides	.	.	.	.	.	.	+	.	+	.

**Companion species with one occurrence**: Poa alpina agg. 21: 1.2; Asplenium trichomanes 21: + ; Cardaminopsis arenosa 21: + ; Potentilla thuringiaca 21: + ; Calamagrostis epigejos 21: + ; Cynosurus cristatus 23: + ; Lactuca serriola 26: + ; Centaurea atropurpurea 26: + ; Lotus corniculatus 28: + ; Chondrilla juncea 28: + ; Pilosella piloselloides subsp. bauhinii 28: + ; Anchusa officinalis 29: 1.3; Apera spica-venti 29: + ; Euphrasia illyrica 30: + ; Agrostis capillaris 32: + ; Silene vulgaris 32: + ; Rubus candicans 32: +

Relevé sites: 21: Obârșia Cloșani—Dealul cu Zgură (5.07.2017); 22–23: Giurgieni—Valea Coșuștei (5.07.2017); 26–31: Rudina (6.07.2017); 32: Seliște—Valea Verde (5.07.2017)

These open grasslands, dominated by true grasses (*Achnatherum calamagrostis*, *Melica ciliata* and *Festuca valesiaca*), are distributed in the submontane belt of the Mehedinți Mts. and precisely, on south-facing, moderately steep slopes with poorly consolidated rendzinic leptosols, usually developed in previously disturbed, abandoned mining sites (30–40 years ago). The soil samples have a near neutral reaction (pH = 6.4–6.6) and a high content of nickel and chrome, similar to those collected in the PA communities (Table 2).

*Asplenium serpentini* and *Alyssum petraeum* are statistically significant discriminant species for AsA with respect to other plant associations (Table 3). Some characteristic species for the order *Halacsyetalia sendtneri* (e.g., *Alyssum murale* subsp. *pichleri*, *Notholaena marantae* and *Stachys recta* subsp. *subcrenata*) are well represented through relatively high frequencies of occurrence (Table 5).

***Sileno saxifragae–Plantaginetum holostei*** ass. nova hoc loco

Holotypus: relevé 38 (Table 6); Abbreviation: SP; Photo: E in Additional file 2: Appendix S2.

**Table 6:** *Sileno saxifragae–Plantaginetum holostei* ass. nova (* holotypus; ser—serpentinites)

Relevé no.	33	34	35	36	37	38*	39	40	41	42
Elevation (m)	1544	1540	1542	1589	1544	1550	1540	1540	1530	1500
Aspect	NE	N	SW	NW	SW	S	S	S	S	SE
Slope (degrees)	5	10	20	10	45	10	10	5	50	55
Total herb cover (%)	65	50	70	50	50	30	35	40	30	50
Plot area (m^2^)	10	10	10	10	10	4	4	4	4	4
Bedrock type	ser	ser	ser	ser	ser	ser	ser	ser	ser	ser
**Diagnostic species at association level**										
Plantago holosteum	2.3	2.3	3.4	2.3	3.4	2.3	2.3	2.4	2.3	3.4
Anthemis carpatica	+	+	+.1	3.3	1.3	+	+	+	1.2	1.2
Silene saxifraga	+	+	.	+	.	+	+	+	+	+
Pilosella rhodopea	+	.	.	.	+	+	+	.	+	+
**Asplenio septentrionalis–Festucion pallentis and Stipo pulcherrimae–Festucetalia pallentis**										
Festuca pseudodalmatica	.	+	+	.	+	.	.	.	.	.
Dianthus henteri	+	+	1.2	.	.	+	.	+	.	.
Festuca pallens	.	.	.	.	.	1	1	1	.	.
Thymus praecox subsp. jankae	.	.	.	+	.	+	.	.	.	.
Saxifraga paniculata	.	+	+	.	.	+	+	.	.	+
Achillea crithmifolia	+	+	+	.	.	.	.	.	.	.
Sedum hispanicum	.	+	.	.	.	+	+	.	.	.
Minuartia verna	.	+	.	.	.	.	.	.	.	.
Stachys recta	.	.	.	+	.	.	.	.	.	.
Asplenium septentrionalis	.	.	.	+	+	.	.	.	.	.
Cerastium pumilum subsp. glutinosum	.	.	.	+	.	.	.	.	.	.
**Festuco–Brometea (incl. Festuco saxatilis–Seslerion bielzii)**										
Festuca saxatilis	2.3	3.4	2.3	1.3	1.2	.	+	.	1.2	1.2
Thymus praecox subsp. polytrichus	+	.	2.2	2.3	.	+	1	+	.	.
Pilosella pavichii	1.2	.	+	.	+	.	+	.	.	+
Jovibarba heuffelii	.	+	+	.	.	+	+	.	+	.
Scabiosa columbaria	+	.	.	+.1	+	.	.	.	.	.
Daphne blagayana	.	+	+	.	.	+	.	.	.	.
Hieracium bifidum	+	+	.	.	.	.	.	.	.	.
Pilosella hoppeana	.	.	+	.	.	.	.	.	.	.
Hypericum perforatum	.	.	+	.	.	.	.	.	.	.
**Seslerion rigidae**										
Scabiosa lucida subsp. barbata	+	.	.	+	.	+	+	.	.	+
Pedicularis comosa	.	+	+	.	.	+	+	.	+	.
Seseli libanotis	.	+	.	.	.	+	.	.	.	+
Iris ruthenica	.	.	.	.	.	+	.	.	.	.
**Juncion trifidi**										
Juncus trifidus	.	.	.	.	+	+	+	+	+	.
Festuca supina	1.2	2.3	+	.	.	.	.	.	+	.
Hypericum transsilvanicum	+	.	+	.	+	+	.	.	.	.
Luzula spicata	.	.	+	.	.	.	.	+	.	.
Phyteuma confusum	.	.	.	.	.	.	.	.	.	+
**Companions**										
Luzula luzuloides	+	+	2.3	+	+	.	.	.	.	+
Campanula rotundifolia	.	+	+	.	+	+	.	.	+	+
Silene lerchenfeldiana	.	1.2	+	.	.	+	+	.	+	+
Deschampsia flexuosa	2.3	+	1.2	+	+.2	.	.	.	.	.
Vaccinium vitis-idaea	+	+	1.2	.	+	.	.	+	.	.
Cytisus nigricans	+	+	+	.	.	.	+	.	.	+
Agrostis capillaris	+.1	1.2	1.2	2.3	.	.	.	.	.	.
Rumex acetosella	+	.	.	.	.	+	.	.	+	+
Genista tinctoria subsp. oligosperma	1.2	.	+	+	.	.	.	.	.	.
Antennaria dioica	1.3	.	+	.	.	.	.	+	.	.
Bruckenthalia spiculifolia	.	.	+	+	.	.	.	+	.	.
Potentilla erecta	+	+	.	.	.	.	.	.	.	.
Sedum annuum	.	+	.	.	+	.	.	.	.	.
Luzula multiflora	.	+	.	.	1.2	.	.	.	.	.
Vaccinium myrtillus	.	.	+	.	+	.	.	.	.	.
Solidago virgaurea	.	.	.	+	.	.	.	.	+	.
Viola declinata	.	.	.	.	+	+	.	.	.	.

**Companion species with one occurrence**: Bellardiochloa variegata 34: + ; Silene nutans subsp. dubia 35: + ; Peucedanum oreoselinum 35: + ; Lotus corniculatus 35: + ; Carlina acaulis 36: + ; Galium album 36: + ; Calamagrostis arundinacea 36: + ; Centaurea stoebe subsp. australis 34: +

Relevé sites: 33–37: Cozia Mts. (19.07.2017); 38–42: Cozia Mts. (7.07.1987)

This plant association includes the saxicolous and heliophilous communities dominated by the widely distributed Mediterranean species–*Plantago holosteum*, and developed in the upper montane belt of the Cozia Mts. (1550*–*1590 m). The vegetation is sparse and its relative cover rarely exceeds 50%. The most common soil type in these sites is dystric leptosol, with a moderately acidic reaction (pH = 4.7–5.6) and a high content of nickel and chrome, closely resembling in this regard the previously described plant associations (Table 2).

Of the numerous (sub)acidophilous taxa, some of them (*Silene saxifraga* and *Pilosella rhodopea*) are good discriminant species and are considered, along with *Anthemis carpatica*, as diagnostic species (Table 3 and 6). A distinctive group is the one including acidophilous species typical for alpine grasslands of the *Juncion trifidi* alliance (Table 6).

Beside the dominant *Plantago holosteum*, a series of neutro-basophilous species (e.g., *Thymus praecox* subsp. *polytrichus, Saxifraga paniculata*) are also represented in the floristic composition of SP, namely those typical for the montane alliance *Seslerion rigidae* and the alpine alliance *Festuco saxatilis-Seslerion bielzii* (Table 6). Of these, *Festuca saxatilis* represents a true differential species of SP with respect to all other ultramafic associations considered (Table 3).

***Minuartio frutescentis–Plantaginetum holostei*** ass. nova hoc loco

Holotypus: relevé 45 (Table 7); Abbreviation: MP; Photo: D in Additional file 2: Appendix S2.

**Table 7:** *Minuartio frutescentis–Plantaginetum holostei* ass. nova (* holotypus; per—peridotites)

Relevé no.	43	44	45*	46	47	48	49	50	51	52
Altitude (m)	432	420	400	360	355	350	430	355	400	360
Aspect	S	SW	SW	SE	E	E	S	SE	S	SW
Slope (degrees)	5	5	10	5	15	10	5	10	5	5
Total herb cover (%)	60	60	45	55	60	50	45	50	55	65
Plot area (m^2^)	10	10	10	10	10	10	10	10	10	10
Bedrock type	per	per	per	per	per	per	per	per	per	per
**Diagnostic species at association level**										
Plantago holosteum	2.3	2.3	2.4	3.4	3.4	3.4	3.3	3.3	2.3	4.5
Minuartia hirsuta subsp. frutescens	2.3	2.3	2.3	2.3	2.2	1.2	+.3	+	3.4	1.3
Anthemis cretica subsp. kitaibelii	+	.	.	+	1.1	+	+	.	+	+
**Asplenio septentrionalis–Festucion pallentis and Stipo pulcherrimae–Festucetalia pallentis**										
Festuca panciciana	2.2	2.2	1.1	1.2	.	.	.	1.2	.	+
Festuca pseudodalmatica	.	+	+	.	.	+	+	.	+	+
Asplenium septentrionale	.	.	.	.	.	.	+	1.2	.	.
Achillea crithmifolia	.	.	.	+	+	+	.	+	+	.
Thymus praecox subsp. jankae	.	.	+	.	.	.	.	.	+	.
Astragalus onobrychis var. linearifolius	+	.	.	.	.	.	.	.	+	+
Alyssum murale	.	+	+	.	.	.	.	+	.	.
Pilosella hoppeana	.	.	.	+	.	+	.	.	.	.
Stachys recta	.	.	.	+	.	.	.	.	+	.
Sempervivum marmoreum	.	.	.	.	.	.	.	+	.	.
**Festuco–Brometea**										
Thymus praecox subsp. polytrichus	+	.	+	2.2	1.3	1.2	1.2	1.3	+.1	1.2
Centaurea stoebe subsp. australis	+	+	+	++.1	+	.	.	.	.	.
Potentilla cinerea	.	.	+	.	+	.	+	+	+	.
Bothriochloa ischaemum	+	.	.	+	+	.	+	.	.	.
Koeleria macrantha	.	+	.	.	.	+	.	.	+	+
Melica ciliata	.	+	.	.	+	.	+	.	.	.
Verbascum glabratum	+	1.1	.	.	.	.	.	+	.	.
Chondrilla juncea	+	.	.	.	+	.	.	.	.	.
Teucrium chamaedrys	.	.	.	.	+	.	.	+	.	.
Silene armeria	.	.	.	.	.	.	.	+	.	+
Asperula cynanchica	.	.	.	.	.	.	.	.	.	+
**Companions**										
Cytisus nigricans	1.2	1.2	1.2	.	.	+	.	.	.	+
Anthoxanthum odoratum	+	+	+	+	.	.	.	.	.	.
Agrostis capillaris	1.2	.	.	+	1.3	.	.	+	.	.
Rumex acetosella	.	+	.	.	+	.	.	.	.	.
Vulpia myuros	.	.	.	+	1.1	.	.	.	.	.
Erigeron canadensis	.	.	.	+	+	.	.	.	.	.

**Companion species with one occurrence**: Danthonia decumbens 43: + ; Pilosella brachiata 44: + ; Carlina vulgaris 47: + ; Scleranthus perennis subsp. dichotomus 51: +

Relevé sites: 43–49: Ohaba de sub Piatră—Vf. Poieni (20.07.2017); 50–52: Vf. Poieni (10.07.1974)

This plant association encompasses the communities dominated by *Plantago holosteum* and *Minuartia hirsuta* subsp. *frutescens*, and developed in the submontane belt of the northern Retezat Mts. The substrates are made up of peridotites, mainly composed of olivine. The soils are represented by superficial, skeletic leptosols with acidic reaction (pH = 4.3–5.1) and low content of nickel and chrome, but high concentration of iron (Table 2). *Anthemis cretica* subsp. *kitaibelii* and *Minuartia hirsuta* subsp. *frutescens* are statistically significant, discriminant taxa against the other plant associations considered (Table 3). By showing a relatively high frequency of occurrence, *Festuca panciciana* is indicated among the diagnostic species of MP (Table 7).

### Indirect ordination of all relevés

**Fig. 3:**
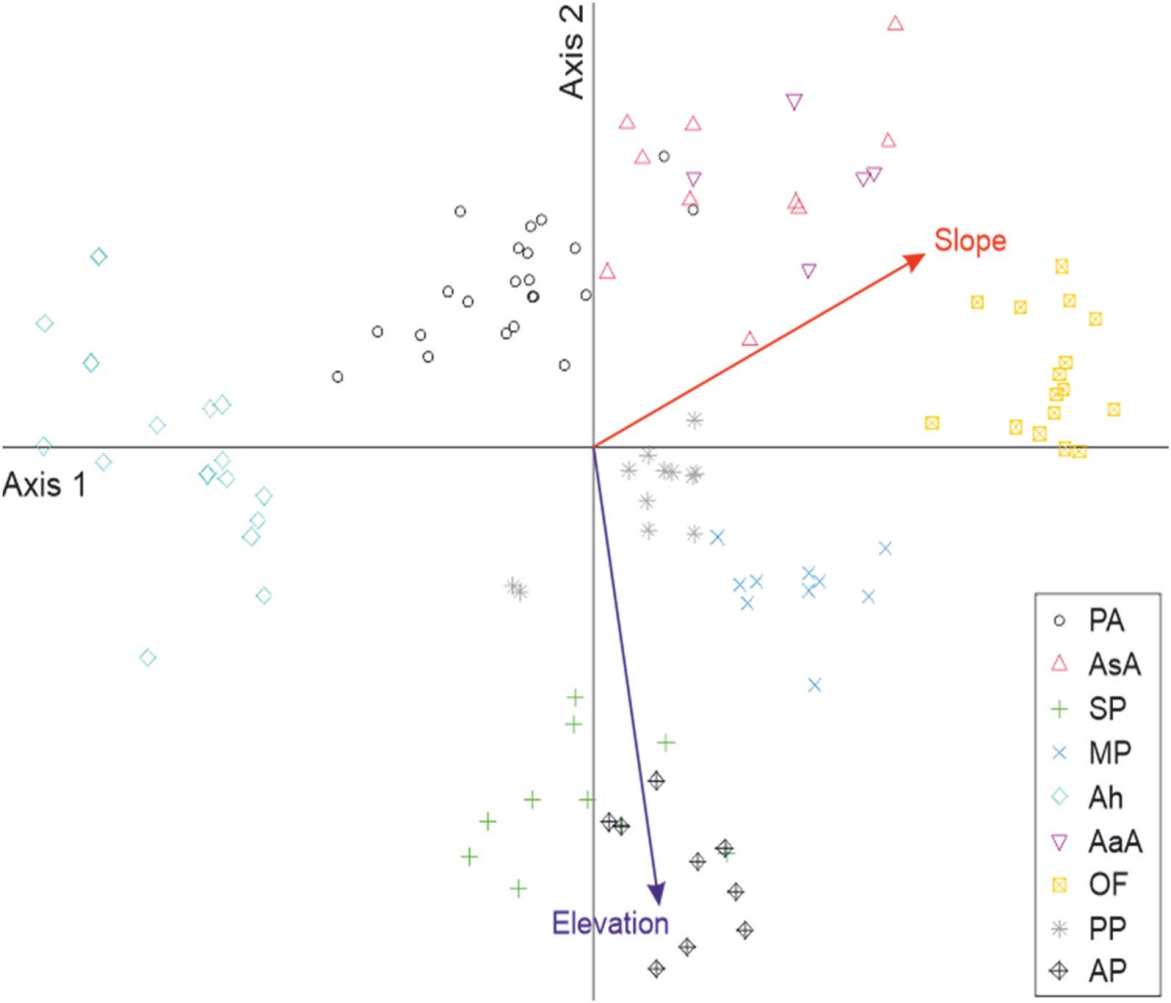
Ordination of the 120 relevés of ultramafic grasslands in the space determined by the two most important NMDS axes, from the total of three axes extracted (final stress = 0.0987; linear R-square = 0.916); the length and orientation of the two environmental vectors stem from the outcome of the linear fitting of elevation and slope against the relevé scores (see Table 8). PA = *Plantago serpentinae–Armerietum halleri*; AsA = *Asplenio serpentini–Achnatheretum calamagrostis*; SP = *Sileno saxifragae–Plantaginetum holostei*; MP = *Minuartio frutescentis–Plantaginetum holostei*; Ah = *Armerietum halleri*; AaA = *Artemisio albae–Achnatheretum calamagrostis*; OF = *Onosmo pavlovae–Festucetum dalmaticae*; PP = *Poo molinerii–Plantaginetum holostei*; AP = *Anthemido–Plantaginetum holostei*

The disposal of relevés along the first NMDS axis reveals a conspicuous differentiation of the Ah and OF communities, which are located at the two extremities (Fig. 3). The species with the largest scores on the first axis are *Deschampsia caespitosa* (at the negative end) and *Petrorhagia prolifera* (at the positive end). The indicator values for soil moisture attributed to these two species are 7 and, respectively 3, suggesting a rainfall gradient overlapping the first NMDS axis. The latter is also significantly, positively related with the slope of the terrain (Table 8, Fig. 3).

**Table 8:** Summary statistics of the independent, linear trend surface fitting of elevation and slope against the relevé scores on the three non-metric multidimensional scaling axes

Response variable	Axis 1	Axis 2	Axis 3	R-square	Prob
Site elevation	0.1416	0.9044	− 0.4024	0.3446	< 0.0001
Terrain slope	0.7922	− 0.4336	0.4294	0.2722	< 0.0001

The relevés of AP and SP are well separated from the others toward the negative end of the NMDS axis 2 (Fig. 3). Among the plant taxa displaying the largest, negative scores on the second axis, there are two calcifugous species (*Cardaminopsis arenosa* subsp. *arenosa* and *Apera spica-venti*), both with the same indicator value for nutrients (6). The second axis is also strongly and negatively correlated with site elevation (Table 8, Fig. 3).

### Dependence of total species richness/cover on topographic variables

**Fig. 4:**
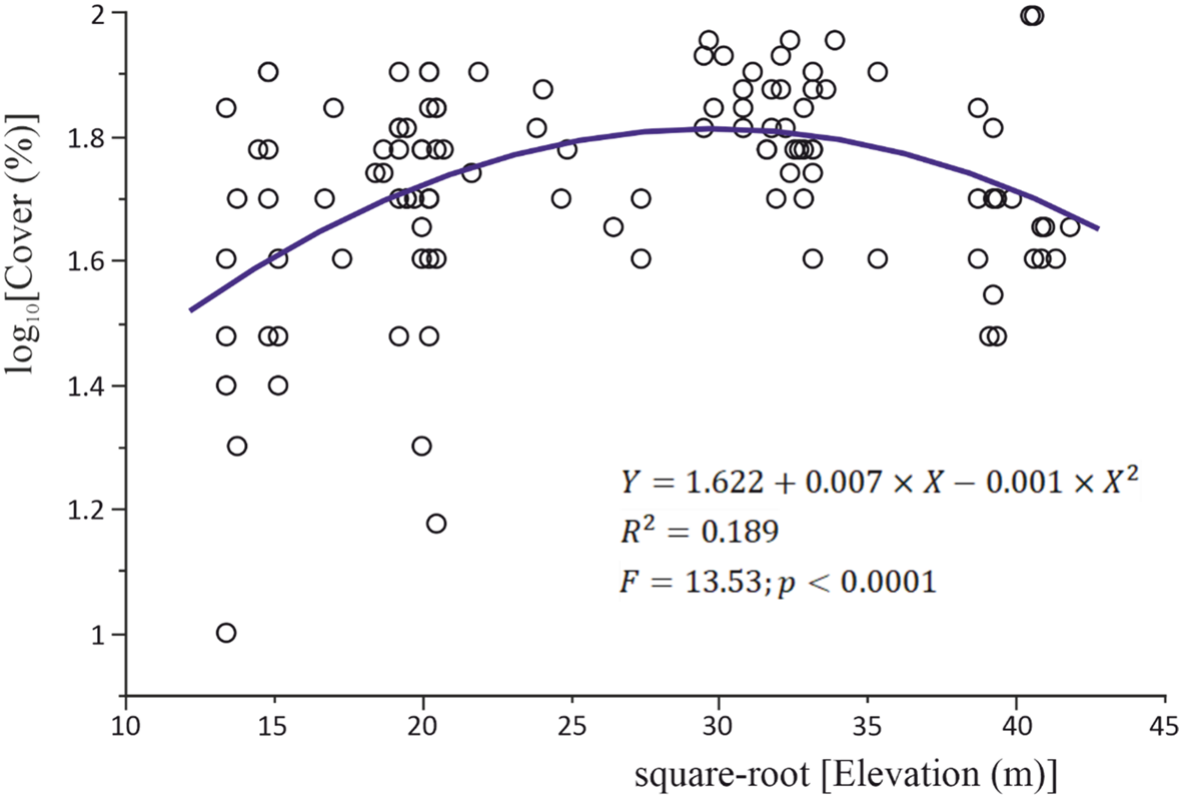
Second-order polynomial regression of total plant cover by site elevation in all ultramafic communities considered (n = 120); all regression coefficients are significantly different from zero (p < 0.0001)

By jointly considering all studied relevés, the total plant cover at the community scale displays a unimodal relationship with site elevation (Fig. 4). The maximum vegetation cover is reached at about 900 m altitude. There is no significant linear or quadratic relationship between total plant cover and terrain slope.

A similar unimodal pattern, but in three-dimensional space, is disclosed by the response of species richness to both elevation and slope, while accounting for the differences in the relevé area (Table 9). The two topographic predictors have confounding effects on species richness, given the significant interaction term in the regression model and their weak but significant positive relationship (Spearman’s rho =  + 0.228; p = 0.0125).

**Table 9:** Raw regression coefficients associated with the significant effects of area, elevation and terrain slope on species richness (all variables involved in the model were either log-transformed, i.e., species number and area, or square root-transformed, i.e. elevation and slope, prior to analysis)

Effect terms	Coefficient estimates	t ratio	Prob >|t|	Model statistics
Intercept	1.113	6.81	< 0.0001	n = 120 R^2^ = 0.687 F = 40.974 p < 0.0001
Area	0.301	4.68	< 0.0001	
Elevation	0.030	5.55	< 0.0001	
Slope	0.119	4.41	< 0.0001	
Elevation × Slope	− 0.011	− 3.60	0.0005	
Elevation × Elevation	− 0.003	− 4.15	< 0.0001	
Slope × Slope	− 0.029	− 3.49	0.0007	

When controlling for the effect of the sampled area, total species cover and richness covary positively, as indicated by their significant partial correlation (Spearman’s rho =  + 0.282; p = 0.0019).

## Discussion

### Regional floristic patterns

The major floristic differentiation of the ultramafic herbaceous communities from central and south-eastern Europe is mainly due to the species of Balkan origin and, to a lesser extent, to regional Carpathian species. Overall, the Ah communities of the order *Violetalia calaminariae* from central Europe are floristically the most distinctive, as they form a separate cluster in correspondence to the first branching of the dendrogram.

All four distinguished Southern Carpathian ultramafic associations (PA, AsA, SP and MP) are assigned to different alliances from *Potentillion visianii* and *Alyssion heldreichii*, which encompass the two western Balkan associations (AaA and PP) and respectively, the central Balkan association (OF) considered in this study. This is fully supported by the separation of the Southern Carpathian and western-central Balkan plant associations in two different large clusters at the second branching of the dendrogram. Phytogeographically, the Southern Carpathian ultramafic communities stand out through the absence of a series of Balkan species (e.g., *Alyssum heldreichii*, *A. markgrafii*, *A. montanum* subsp. *serbicum*, *Euphorbia glabrifolia*, *Linaria rubioides*, *Potentilla visianii*, *Stachys scardica* and *Stipa novakii*) and especially, the presence of several regional endemic taxa: *Dianthus giganteus* subsp. *banaticus* (in PA and AsA), *Hypericum transsilvanicum, Dianthus henteri, Scabiosa lucida* subsp. *barbata*, *Genista tinctoria* subsp. *oligosperma*, *Viola declinata* and *Silene nutans* subsp. *dubia* (in SP), and *Anthemis cretica* subsp. *kitaibelii* (in MP).

Many east-submediterranean species spread northward along the Balkan-Carpathian connection prior to partial blocking by the Danube River in late Miocene (Jakovljević et al. 2011; Hurdu et al. 2012; Knežević and Ganić 2013). Therefore, the occurrence of several Balkan species (e.g., *Alyssum murale*, *Asplenium serpentini*, *Bromus pannonicus*, *B. riparius*, *Notholaena marantae*, *Scleranthus perennis* subsp. *dichotomus*, *Silene bupleuroides* subsp. *staticifolia* and *Stachys recta* subsp. *subcrenata*) in the ultramafic communities (PA and AsA) from the south-western end of the Carpathians (i.e., the Mehedinți Mts.) allowed for their assignation into the order *Halacsyetalia sendtneri*, despite the absence of the western Balkan, endemic serpentinophyte—*Halacsya sendtneri*. These floristic affinities are undoubtedly facilitated by the neutral reaction (pH) of serpentinite soils from the Mehedinți Mts., which closely matches the edaphic properties encountered on ultramafic substrates in the western Balkan Peninsula (Tatić 1969; Bergmeier et al. 2009; Tzonev et al. 2013).

A special mention deserves the unexpected grouping of the (south-Balkan) AP communities together with the (Southern Carpathian) SP and MP ones in the dendrogram, which reflects certain compositional affinities. These are mainly determined by the presence of *Plantago holosteum* and several subacidophilous species (e.g., *Anthemis carpatica, Pilosella hoppeana* subsp. *macrantha, Rumex acetosella, Luzula multiflora*) that are common to AP and SP/MP, as their habitats share similar edaphic conditions in terms of topsoil reaction. Nevertheless, the AP communities stand out through a series of species characteristic for the alliance *Trifolion parnassi*, like *Astragalus thracicus* subsp. *parnassi, Leontodon stenodon, Dianthus viscidus, Festuca macedonica* and *Minuartia recurva* subsp. *condensata* (Quézel 1967).

On ultramafic substrates, there is a tendency for the number of serpentinophytes to decrease toward higher latitudes or elevations, probably related to the more humid climate. For instance, there is a conspicuous difference in the number of characteristic species for *Violetalia calaminariae* (central Europe) *versus Halacsyetalia* and *Astragalo–Potentilletalia* (submediterranean Balkans). Unfortunately, the data in hand do not allow an appropriate testing of this hypothesis, which has some support from previous studies reporting that a larger amount of precipitations combined with a lower soil pH can lead to substantial leaching of Ni and consequently, to mitigation of its toxic effects (Chardot et al. 2007; D’Amico and Previtali 2012).

### Syntaxonomical and nomenclatural aspects

None of the alliances composing the order *Halacsyetalia*, as acknowledged by Mucina et al. (2016), could be employed for the syntaxonomical assignation of the PA and AsA associations, given the obvious floristic dissimilarities. The description of a new alliance, which should include the two mentioned plant associations, cannot be supported due to the very low number of serpentinophytes in our study area, compared with the central and western Balkans. The most appropriate assignation proved to be to the alliance *Thymion jankae* nom. inval. (art. 2b in Theurillat et al. 2021) that was proposed by Kojić et al. (1992) as part of the Balkan order *Halacsyetalia*, but without providing a holotypus. Therefore, in accordance with the art. 6 in Theurillat et al. (2021), we validated the mentioned alliance by providing a holotypus, that is the association *Poo alpinae–Plantaginetum holostei* Kojić et Ivanović 1953. In order to complete the top-to-bottom typification of the syntaxa, we herein designate a lectotypus (relevé 1 in Table 4 in Cincović and Kojić (1956)) of the previously mentioned association. The core area of distribution of the alliance *Thymion jankae* of ultramafic vegetation lies in the Serbian-Bosnian Dinarides, whereas the smaller Southern Carpathian area represents a northeastern disjunction.

The SP association was long ago delineated under the name *Anthemido carpaticae–Plantaginetum holostei* (Coldea and Pop 1988), but it proved to be invalid (art. 31 in Theurillat et al. 2021) given that a homonymous syntaxon had been previously described by Raus (1987) in the Ossa Mts. (central Greece).

### Synecological patterns

Soil moisture is the most important ecological gradient along which major changes in the species composition of the studied ultramafic communities can be observed. This complex gradient is very likely determined, among other factors not considered here (e.g., soil texture), by the variation in rainfall and water runoff that stem from different mesoclimatic conditions (temperate to submediterranean) and especially, topographic conditions (colline to upper montane belt and, flat terrain to very steep slopes). In this respect, the OF (submediterranean, steep-sloped) *versus* Ah (temperate, mild-sloped) communities, as well as the AsA, PA and AaA (colline) *versus* SP and AP (montane) coenoses, are positioned at the two ends of the moisture gradient. Within the Southern Carpathians there is no sharp differentiation among all single syntaxa but, after their grouping by altitudinal belt, a clear ordination from xeric (AsA and MP) to mesic (PA and SP) communities is noticeable.

A weak and not steady increase in soil acidity overlaps the long altitudinal gradient. Consequently, the most acidic soils are encountered in SP and AP coenoses that are distributed at the highest elevations. At the opposite end of the gradient, the AsA, PA and AaA communities developing on neutral soils are disposed. Considering that under humid conditions soil characteristics usually diverge from those developed in xeric climates but similar parental material (D’Amico et al. 2014), the inferred positive relationship between soil acidity and elevation can be partly explained by stronger leaching of base cations with increasing precipitations toward higher altitudes.

The revealed synecological patterns point to the major importance of topographic conditions in driving the variation in species composition. This outcome is in accordance with other studies reporting that physiographic variables and base nutrients (N, K, P, Ca) are more important than heavy metal concentrations in explaining the vegetation—environment relationships on ultramafic substrates (Chiarucci et al. 1998a, 1998b, 2001; Tsiripidis et al. 2010; D’Amico et al. 2014; El Ghalabzouri et al. 2015).

The particular floristic composition of the SP communities discloses two striking features related to the coexistence of species with contrasting ecological requirements: acidophile *versus* neutro-basiphile and, xero-thermophile *versus* meso-orophile. The former is probably related to the heterogeneous nature of the olivine-rich, ultramafic substrates from the Cozia Mts. (Hann and Szász 1981). The latter is due to the upslope shift in the distribution of xero-thermophilous species on sunny, steep, rocky habitats and, the downslope spread of microthermal-alpine species in valleys or depressions affected by inversions of the thermal lapse rate (Coldea and Pop 1988).

### Total species cover/richness at community scale

Apparently unexpected, the total species cover reaches its maximum in the lower montane belt, that is in correspondence with intermediate amounts of rainfall. While at lower elevations the limiting factor is definitely the water deficit, the poorer vegetation cover at higher elevations is probably due to the steeper, rockier slopes. Another possible explanation could be the negative effect of Ni availability on vegetation cover at lower soil pH (and implicitly, at higher elevations), as reported by several authors (Robinson et al. 1996; Chiarucci et al. 1998b; Tsiripidis et al. 2010).

At the community scale, plant species richness is much more predictable than total plant cover with respect to site conditions, as indicated by the high proportion of variance explained in the multiple regression. The unimodal response of species richness to both elevation and slope is also related to the negative effects of water and nutrient deficit at low elevations/on steep slopes and respectively, at higher elevations. However, the low species richness observed on flat or gently inclined terrain cannot be understood without taking into account that mild-sloping habitats are located at low elevations. It seems that overall, topographic conditions are more important than soil nutrients also in driving species richness on ultramafic substrates. Reddy et al. (2009) reached a similar conclusion when observing that soil chemistry does not play a significant role in determining plant diversity in serpentine areas of the Witwatersrand ranges (South-Africa).

Although probably less important in case of open, herbaceous, ultramafic vegetation, we cannot exclude the contribution of several distinct phenomena to the peak of species richness at intermediate elevations i.e., the mid-domain effect (Colwell and Lees 2000), the intermediate disturbance effect (Connell 1978) and the ecotone effect (Odum 1971) at the interface between the colline and montane vegetation belts. In fact, such possible effects were invoked by Dubuis et al. (2011) for explaining a similar hump-shaped pattern of species richness along the elevational gradient in southern Swiss Alps and precisely, in open, non-woody vegetation sampled at a comparable scale (4 m^2^) but developed on non-ultramafic substrates.

The congruent responses of species cover and richness with respect to elevation seem to indicate that the number of species is roughly largest in sites with high vegetation cover, a pattern observed as well in the Californian ultramafic sites (Harrison et al. 2006). This obviously denotes that no competitive exclusion occurs even under more favourable moisture conditions.

## Conclusions and limitations

The syntaxonomic distinction of four ultramafic grassland types in the Southern Carpathians is well supported on the basis of their overall species composition, although they host few differential species with respect to their Balkan Peninsula counterparts. In this respect, *Sileno–Plantaginetum holostei* stands out as the best individualised syntaxon.

Given that the topographic conditions are precursors of, but closely related to soil moisture and fertility, we conclude that the species composition, total cover and richness in all studied ultramafic grassland communities are largely driven by site elevation and slope, and to a lesser extent by soil nutrients. To our knowledge, the present study is the first to disclose unimodal relationships between total species cover/richness and local physiographic variables in ultramafic herbaceous communities.

Finally, we must acknowledge some inherent limitations in our study, given that: (i) soil moisture and, partially, soil acidity were inferred as latent (not directly measured) gradients, (ii) the content of some soil macronutrients (e.g., N and P) was not considered in our analyses, and (iii) the number of relevés pertaining to each plant association was relatively low, due to either low availability of published data or limited extension of serpentine areas in the Southern Carpathians.

## Supplementary Information

**Additional file 1: Appendix S1.** Synoptic table displaying the species frequencies (%) of occurrence in several ultramafic herbaceous communities from central Europe (columns 1–2), Southern Carpathians (colums 3–6), western Balkans (columns 7–8), central Balkans (column 9) and southern Balkans (column 10). Values in bold (within grey-shaded cells) correspond to the regional differential species in the Southern Carpathians. Abbreviations: Ah = *Armerietum halleri*; DA = *Diantho gratianopolitanae–Armerietum halleri*; PA = *Plantago serpentinae–Armerietum halleri*; AsA = *Asplenio serpentini–Achnatheretum calamagrostis*; SP = *Sileno saxifragae–Plantaginetum holostei*; MP = *Minuartio frutescentis–Plantaginetum holostei*; AaA = *Artemisio albae–Achnatheretum calamagrostis*; PP = *Poo molinerii–Plantaginetum holostei*; OF = *Onosmo pavlovae–Festucetum dalmaticae*; AP = *Anthemido–Plantaginetum holostei*.

**Additional file 2: Appendix S2.** Photos of the studied serpentine vegetation in the South-Eastern Carpathians (Romania). (A) Thermophilous phytocoenosis from the Mehedinți Plateau, with *Asplenium serpentini* and *Notholaena marantae* (photo: I. Ciortan, 04.07.2017). (B) and (C) Typical serpentine plant species from the Mehedinți Mts., showing *Plantago serpentina* and respectively, *Armeria halleri* (photo: I. Ciortan, 05.07.2017). (D) Open communities dominated by *Plantago holosteum*, *Anthemis cretica* subsp. *kitaibelii* and *Minuartia frutescens* on antigorite-rich rocks from the northern Retezat Mts., Poieni Peak—Ohaba de sub Piatră (photo: M. Ciobanu, 21.07.2017). (E) Phytocoenosis featuring *Plantago holosteum*, *Brukenthalia spiculifolia* and *Anthemis carpatica* from the Cozia Mts. (photo: P.M. Szatmari, 19.07.2017).

## Data Availability

The datasets collected and analysed during the current study are available from the first author (GC, gheorghe.coldea@icbcluj.ro) on reasonable request.
