# Correction: Southern Carpathian ultramafic grasslands within the central-southeast European context: syntaxonomic classification and overall eco-coenotic patterns

**DOI:** 10.1186/s40529-022-00362-9

**Published:** 2022-11-21

**Authors:** Gheorghe Coldea, Dan Gafta, Gavril Negrean, Adrian Ilie Stoica, Bogdan-Iuliu Hurdu

**Affiliations:** 1 Institute of Biological Research, National Institute for Research and Development in Biological Sciences, 48 Republic Street, Cluj-Napoca-Napoca, Romania; 2 Department of Taxonomy and Ecology and Centre 3B, Babe-Bolyai University, 42 Republic Street, Cluj-Napoca-Napoca, Romania; 3 Dimitrie Brândză Botanic Garden, 32 Cotroceni Road, Bucharest, Romania

## Correction: Botanical Studies (2022) 63:29 https://doi.org/10.1186/s40529-022-00355-8

In the original publication of the original article (Coldea et al. 2022), the authors identified the following error.

Following further nomenclatural inquiries on old, not readily available papers, we are constrained to reconsider the validation of both the alliance Thymion jankae and the association Poo alpinae-Plantaginetum carinatae.

First, we attempted to validate the alliance Thymion jankae (pages 15–16) based on the claim of Kuzmanović’s et al. (2016) according to which the mentioned alliance had not been validly published, since the nomenclatural type was not explicitly indicated. Nevertheless, in the original diagnosis in Kojić et al. (1992) no other association than Poo alpinae-Plantaginetum carinatae was mentioned to pertain to the alliance Thymion jankae and thus, there was no need to indicate a type (Art. 5a in Theurillat et al. 2021). Therefore, the alliance was fully defined and validly published in Kojić et al. (1992), and should be referred as Thymion jankae Kojić et Mrfat‐Vukelić in Kojić et al. (1992).

Second, the proposed lectotypus of Poo alpinae-Plantaginetum carinatae Kojić et Ivanović 1953 (page 16) should be actually considered a neotypus, as none of the original relevés published in Kojić and Ivanović (1953) can be declared as a type in the absence of the taxon (Thymus praecox) giving the name to the above alliance. In fact, only a generic Thymus sp. appears in the Table 3 in Kojić and Ivanović (1953).
