# Supplementary material for: Southern Carpathian ultramafic grasslands within the central-southeast European context: syntaxonomic classification and overall eco-coenotic patterns
Source: Bot Stud. 2022 Oct 12;63:29. doi: 10.1186/s40529-022-00355-8 (PMC9556682; doi:10.1186/s40529-022-00355-8)

**Appendix S2**

Photos of the studied serpentine vegetation in the South-Eastern Carpathians (Romania).

(A) Thermophilous phytocoenosis from the Mehedinți Plateau, with *Asplenium serpentini* and *Notholaena marantae* (photo: I. Ciortan, 04.07.2017).

(B) and (C) Typical serpentine plant species from the Mehedinți Mts., showing *Plantago serpentina* and respectively, *Armeria halleri* (photo: I. Ciortan, 05.07.2017).

(D) Open communities dominated by *Plantago holosteum*, *Anthemis cretica* subsp. *kitaibelii* and *Minuartia* *frutescens* on antigorite-rich rocks from the northern Retezat Mts., Poieni Peak – Ohaba de sub Piatră (photo: M. Ciobanu, 21.07.2017).

(E) Phytocoenosis featuring *Plantago holosteum*, *Brukenthalia spiculifolia* and *Anthemis carpatica* from the Cozia Mts. (photo: P.M. Szatmari, 19.07.2017).


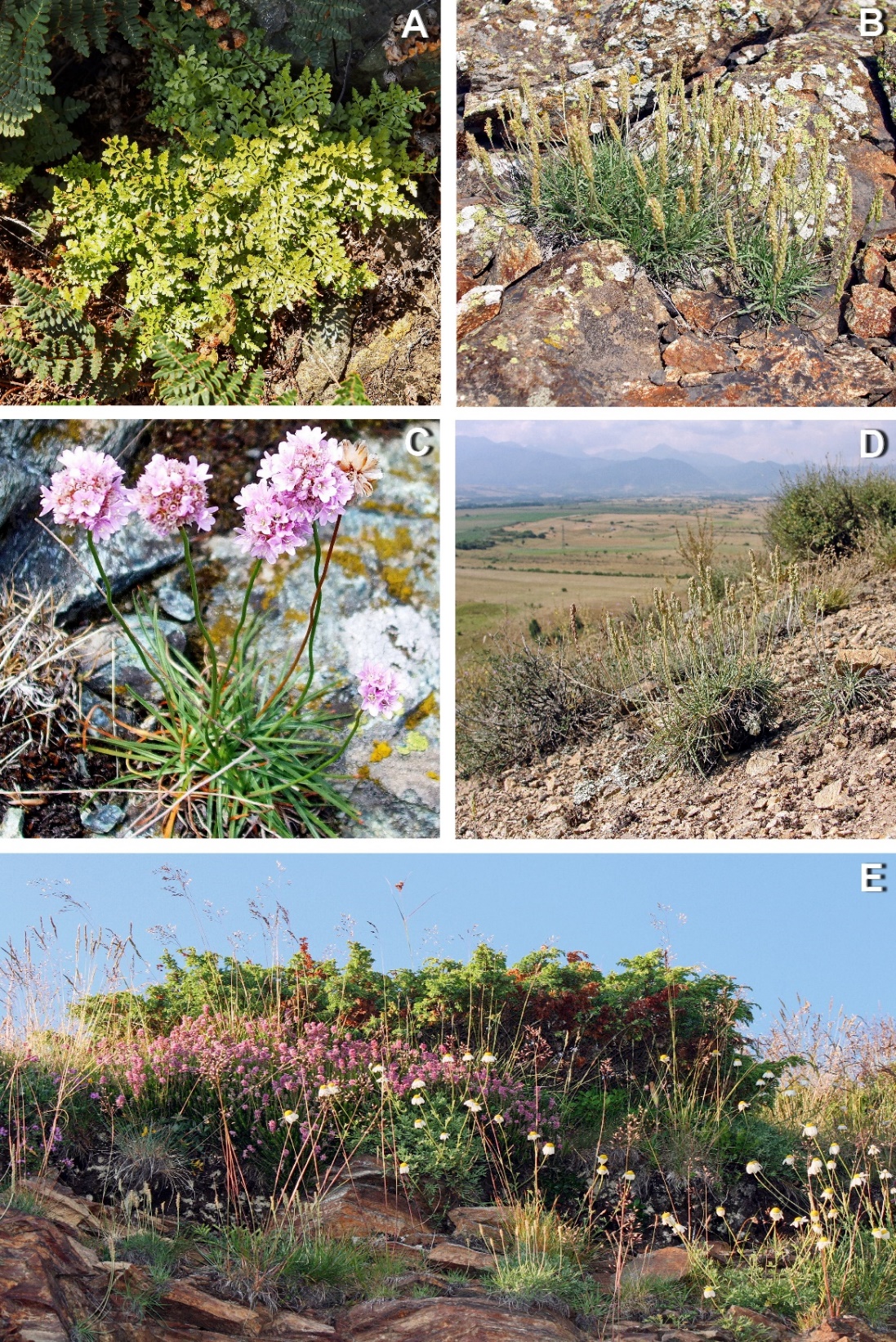

Supplement: Supplementary file 2 — Additional file 2: Appendix S2. Photos of the studied serpentine vegetation in the South-Eastern Carpathians (Romania). (A) Thermophilous phytocoenosis from the Mehedinți Plateau, with Asplenium serpentini and Notholaena marantae (photo: I. Ciortan, 04.07.2017). (B) and (C) Typical serpentine plant species from the Mehedinți Mts., showing Plantago serpentina and respectively, Armeria halleri (photo: I. Ciortan, 05.07.2017). (D) Open communities dominated by Plantago holosteum, Anthemis cretica subsp. kitaibelii and Minuartia frutescens on antigorite-rich rocks from the northern Retezat Mts., Poieni Peak—Ohaba de sub Piatră (photo: M. Ciobanu, 21.07.2017). (E) Phytocoenosis featuring Plantago holosteum, Brukenthalia spiculifolia and Anthemis carpatica from the Cozia Mts. (photo: P.M. Szatmari, 19.07.2017). [file 40529_2022_355_MOESM2_ESM.docx]
